# Radiomics-based prediction of response to immune checkpoint inhibitor treatment for solid cancers using computed tomography: a real-world study of two centers

**DOI:** 10.1186/s12885-022-10344-6

**Published:** 2022-11-30

**Authors:** Yang Yu, Yuping Bai, Peng Zheng, Na Wang, Xiaobo Deng, Huanhuan Ma, Rong Yu, Chenhui Ma, Peng Liu, Yijing Xie, Chen Wang, Hao Chen

**Affiliations:** 1 The Department of Tumor Surgery, Lanzhou University Second Hospital, Lanzhou, 730030 Gansu China; 2 The Second Clinical Medical College, Lanzhou University, Lanzhou, 730000 Gansu China; 3 Department of MR, Lanzhou University Second Hospital, Lanzhou, 730030 Gansu China; 4 Department of Radiology, Gansu Provincial Cancer Hospital, Lanzhou, 730050 Gansu China; 5 Department of Radiology, Lanzhou University Second Hospital, Lanzhou, 730030 Gansu China; 6 Department of General Surgery, Lanzhou University Second Hospital, Lanzhou, 730030 Gansu China

**Keywords:** Radiomics, immune checkpoint inhibitor, marker, predictive model, response

## Abstract

**Background:**

Immune checkpoint inhibitors (ICIs) represent an approved treatment for various cancers; however, only a small proportion of the population is responsive to such treatment. We aimed to develop and validate a plain CT-based tool for predicting the response to ICI treatment among cancer patients.

**Methods:**

Data for patients with solid cancers treated with ICIs at two centers from October 2019 to October 2021 were randomly divided into training and validation sets. Radiomic features were extracted from pretreatment CT images of the tumor of interest. After feature selection, a radiomics signature was constructed based on the least absolute shrinkage and selection operator regression model, and the signature and clinical factors were incorporated into a radiomics nomogram. Model performance was evaluated using the training and validation sets. The Kaplan–Meier method was used to visualize associations with survival.

**Results:**

Data for 122 and 30 patients were included in the training and validation sets, respectively. Both the radiomics signature (radscore) and nomogram exhibited good discrimination of response status, with areas under the curve (AUC) of 0.790 and 0.814 for the training set and 0.831 and 0.847 for the validation set, respectively. The calibration evaluation indicated goodness-of-fit for both models, while the decision curves indicated that clinical application was favorable. Both models were associated with the overall survival of patients in the validation set.

**Conclusions:**

We developed a radiomics model for early prediction of the response to ICI treatment. This model may aid in identifying the patients most likely to benefit from immunotherapy.

**Supplementary Information:**

The online version contains supplementary material available at 10.1186/s12885-022-10344-6.

## Background

Recent research has demonstrated that immunotherapy with immune checkpoint inhibitors (ICIs) is significantly effective in patients with various types of cancer. Several ICIs, including anti-programmed cell death protein–1 (PD1) and anti-programmed cell death protein ligand–1 (PDL1) antibodies, have been approved as treatments for solid cancers, such as lung, liver, gastrointestinal, bladder, and kidney cancers and melanoma [1–3]. Although ICIs have shown promise in the context of cancer management, some challenges with optimizing their benefits remain. Notably, the response to ICI treatment is often low in clinical environments, highlighting the need to identify patients most likely to benefit from ICIs, to reduce risks and maximize efficiency.

At present, several research groups have focused on developing effective predictors of ICI response. Some studies have identified clinical characteristics such as body mass index (BMI), sex, and age as promising predictors given their association with the response to immunotherapy [4–6]. However, predictors derived from the tumor microenvironment remain the most accepted indicators. In clinical practice, tumor PDL1 expression, microsatellite instability status, and tumor mutational burden are commonly recommended as criteria for guiding the selection of patients for immunotherapy [7–10]. Despite their important roles in cancer immunotherapy, these criteria have exhibited varying predictive performance across different cancer types and different degrees of invasiveness, limiting their clinical utilization.

Radiomics is an emerging technique that can extract quantitative high-dimensional data from medical images [11]. Studying tumor-derived data generated via radiomics provides crucial information related to tumor biology. With the radiomics data, novel imaging biomarkers could be developed for patient classification and prediction of treatment response, which makes a better implementation of personalized management in cancer [12]. As the response to immunotherapy highly relies on the tumor microenvironment, it will be possible to make an early prediction by using the radiomics features linked to the tumor biology. To examine the feasibility of predicting immunotherapy outcomes using radiomics-based tools, we conducted a real-world study on ICI-treated patients. In this study, we developed and validated a plain CT-based model for predicting the response to ICI treatment in patients with solid cancers and explored the clinical significance of the constructed model, intending to provide support for personalized cancer treatment.

## Methods

**Fig. 1:**
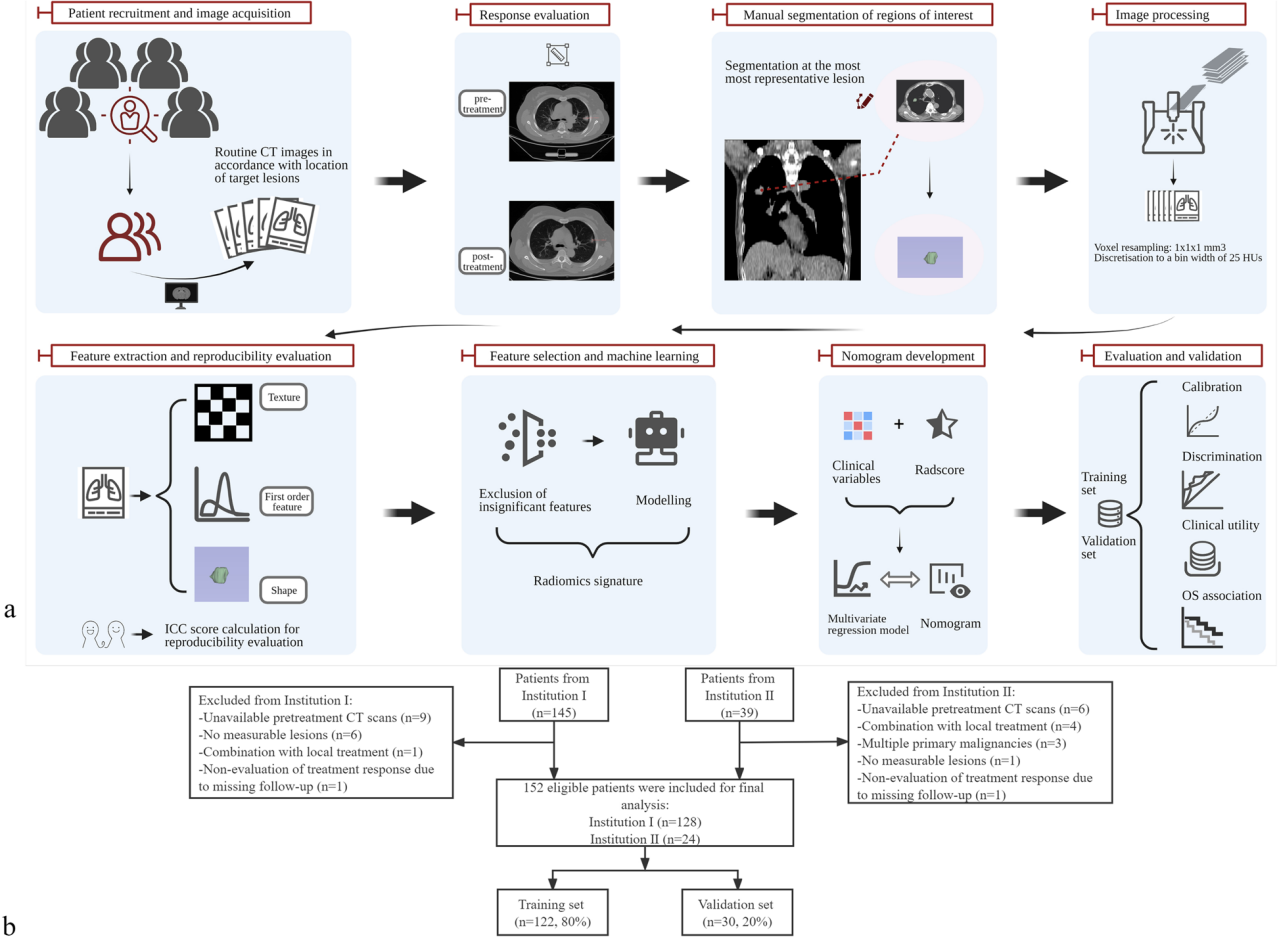
Study workflow (**a**) and patient selection process (**b**). HUs: Hounsfield units; ICC: Inter- and intra-class correlation coefficient; Radscore: radiomics score; OS: overall survival

The study design and basic workflow are illustrated in Fig. 1a.

### Patients and data collection

This retrospective study was approved by the Institutional Research Ethics Committee of our hospital and the requirement for informed consent was waived from patients. Patients who received ICIs at two medical centers (Institution I and Institution II) from October 2019 to October 2021 were included. Inclusion criteria were as follows: (1) diagnosis of solid cancer and receipt of ICIs as first- or later-line treatment, (2) availability of complete clinical data, (3) availability of CT images obtained within 14 days before ICI treatment, and (4) at least one CT re-evaluation within 6 months after treatment. The exclusion criteria were as follows: (1) receipt of concurrent or subsequent local combination treatments, (2) presence of multiple primary malignancies, (3) absence of measurable lesions based on the Response Evaluation Criteria in Solid Tumors (RECIST) 1.1 [13], and (4) previous immunotherapy. The data of the enrolled patients were divided into training and validation sets at a 4:1 ratio using stratified random sampling. The following clinical data were obtained from medical records: sex, age, BMI, tumor type, clinical stage, number of metastases (defined as the number of metastatic regions per patient), and treatment information (e.g., ICI type, combination therapy, and line of therapy).

### Outcome evaluation

The treatment response defined based on the RECIST was used as the patient label to train the models. A dichotomous response status was used to label patients as responders or non-responders. Each patient was evaluated for a response during the first CT follow-up, which was performed at least 1 month after the initiation of treatment, to ensure sufficient follow-up time and evaluate consistency. Patients who exhibited a complete or partial response according to RECIST 1.1 were considered “responders,” while those who exhibited stable or progressive disease after treatment were considered “non-responders.” Overall survival (OS) was also assessed during follow up. In this study, OS was defined as the time from ICI initiation to death from any cause or the last follow up in surviving patients.

### Acquisition of CT images

CT scans obtained within 14 days before treatment were used for analysis. Plain CT scans were acquired using spiral scanners (GE Medical Systems, Philips, and Siemens) and reconstructed in the axial plane using a tube voltage of 100–120 kVp, slice thickness of 1–8 mm, and pixel spacing of 0.62–0.98 mm. Further details concerning the imaging reconstruction parameters are included in Additional file Table S1.

### Tumor segmentation and extraction of radiomics features

Tumor segmentation was performed using 3D slicer software (version 4.11.20210226, https://www.slicer.org). Two experienced radiologists segmented the regions of interest by manually delineating the boundaries of the target lesion slice by slice. The most representative lesion was selected as the region of interest for segmentation to ensure a single lesion per patient. CT images were pre-processed before feature extraction. All CT images and the corresponding segmentation data were normalized by resampling to a pixel size of 1 × 1 × 1 mm^3^ and discretized to a bin width of 25 Hounsfield units (HUs). In addition to the original images, wavelet-transformed images were used for feature extraction. During wavelet transformation, high- and low-pass filters were applied on the x, y, and z axes to produce eight decomposition images, which were labeled as LLL, LLH, LHL, LHH, HLL, HLH, HHL, and HHH. L and H represented low- and high-frequency signals in each direction, respectively. No limitations on feature classes were set for each extraction. Subsequently, 851 features (107 original features and 744 wavelet features) were extracted from the plain CT images using an open-source Python package: Pyradiomics (https://pyradiomics.readthedocs.io/en/latest/index.html, version 3.0.1, Computational Imaging & Bioinformatics Lab, Harvard Medical School). The Pyradiomics adheres to the Imaging Biomarker Standardization Initiative (IBSI) for the most of part. Z-score normalization with the same mean and standard deviation were performed to the extracted features in both the training and validation sets.

### Intra-observer and inter-observer reliability of the radiomics features

Thirty randomly selected CT images were segmented by two radiologists during the same period to assess inter-observer reliability. Intra-observer reliability was assessed by analyzing de novo segmentations of 20 randomly selected images performed by one radiologist after a 2-week interval. The same radiologist performed the remaining image segmentations. Intra-class correlation coefficients (ICCs) were calculated for each feature to determine inter- and intra-observer agreement, with values > 0.75 denoting good agreement. Only features with good inter- and intra-observer agreement were retained.

### Feature selection and construction of the radiomics signature

The feature selection and model construction were performed by using the treatment response we defined during outcome evaluation. The Student’s t-test was first performed for each extracted feature with ICCs > 0.75 to identify the features that differed significantly between responders and non-responders. Levene’s test was used to assess the equality of variances before performing t-tests. For the features that didn’t meet a normal distribution, Mann–Whitney U test was used for identification of differential features. Significant features (*p* < 0.05) based on the results of t-test/Mann–Whitney U test were maintained for further selection. In the training set, all significant features were entered into a least absolute shrinkage and selection operator (LASSO) regression algorithm (α = 1) to select the most predictive features with nonzero coefficients. The tuning regularization parameter, λ, was determined using 10-fold cross-validation based on the minimum criteria. As different types of cancer were included in this study, the location of the target lesions was also used as a variable in these analyses to eliminate the influence of organ heterogeneity on textural patterns. The radiomics signature was constructed by linearly combining the selected features with their nonzero coefficients. Subsequently, a radiomics score (radscore) was calculated for each patient based on the radiomics signature.

### Development of a radiomics-based nomogram

Univariate logistic regression analysis was performed on the potential predictors of treatment response, including the clinical factors and radscore, for the overall population. Variables exhibiting a significant association (*p* < 0.05) with treatment response in the univariate analysis were used to develop a multivariate logistic regression model in the training set. A radiomics-based nomogram was constructed using the radscore and clinical factors to visualize the individualized results of this model. A nomogram score (nomoscore) was calculated for each patient based on a linear combination of selected variables and their coefficients obtained from the multivariate regression analysis.

### Evaluation of predictive performance and model validation

Hosmer–Lemeshow H test was used to evaluate the calibration of the two models (radscore and nomogram) based on the training and validation data. The area under the curve (AUC) of the receiver operating characteristic (ROC) curve was used to evaluate discriminative ability. Difference between two ROC curves was tested using the DeLong’s method. The sensitivity, specificity, and accuracy of the two models were calculated for both datasets based on the optimal cut-off values determined from the training set by maximizing the Youden index. The clinical utility of the models was evaluated using decision curve analysis, which can quantify the net benefits of models at different threshold probabilities [14].

### Exploration of the associations between established models and patient outcomes

The radscores and nomoscores were compared between responders and non-responders, and the differences between the groups were visualized using a boxplot. Survival analysis grouped by the radscores and nomoscores was performed for patients in the validation set to clarify the association between each model and OS.

### Statistical analysis

All statistical analyses were performed using R (version 4.0.2; https://www.r-project.org) and Python (version 3.6.5; https://www.python.org). Continuous data are presented as the mean ± standard deviation, while categorical data are presented as numbers with percentages. The Student’s t-test was used to analyze normally distributed quantitative data, and Levene’s test was used to assess the equality of variances. The Mann–Whitney U-test was used to analyze the non-normally distributed data. Chi-square tests and Fisher’s exact tests were used to compare categorical data. Survival curves were plotted using the Kaplan–Meier method, and the difference between the two curves was assessed using the log-rank test. A Cox proportional hazards model was used to calculate the hazard ratio for OS. All statistical tests were two-sided, and statistical significance was set at *p* < 0.05. The following external R packages were used: (1) the “irr” package for intra-class correlation coefficient score calculation; (2) the “glmnet” package for LASSO logistic regression; (3) the “pROC” and “ROCR” packages for ROC analysis and DeLong’s test; (4) the “rms” package for the nomogram; (5) the “ResourceSelection” package for the Hosmer–Lemeshow H test; (6) the “ggDCA” package for decision curve analysis; and (7) the “survival” and “survminer” packages for survival analysis.

## Results

### Patient characteristics

A total of 152 patients were included in this study, including 128 from institution I and 24 from institution II. During patient selection, 32 patients were excluded for the following reasons: unavailable pretreatment CT scans, combination with local treatment, multiple primary malignancies, no measurable lesions, and non-evaluation of treatment response due to missing follow-up. Using randomization, the data of 122 patients were allocated to the training set, while those of 30 patients were allocated to the validation set. The details are shown in Fig. 1b.

Among the included patients, 119 were men, while 33 were women. The mean age and BMI (±standard deviation) were 59.62 ± 10.07 years and 22.74 ± 3.52 kg/m^2^, respectively. Five types of cancer were observed, the most prevalent of which was lung cancer (*n* = 78), followed by gastric cancer (*n* = 39), hepatocellular carcinoma (*n* = 16), esophageal cancer (*n* = 13), and colorectal cancer (*n* = 6). Most patients had advanced disease (stage III/IV, *n* = 143), with the number of metastases ranging from one to five. Six types of target lesions were employed for data analysis, including those of the esophagus, liver, lung, stomach, lymph nodes, and others. Of these patients, **34.9%** (*n* = 53) showed a clinical response to immunotherapy, whereas 65.1% (*n* = 99) did not. The patient treatment characteristics and detailed information are listed in Table 1.

**Table 1:** Characteristics of included patients

Characteristics		Overall
n		152
Sex (%)	Male	119 (78.3)
	Female	33 (21.7)
Age, mean (SD)		59.62 (10.07)
BMI, mean (SD)		22.74 (3.52)
Tumor type (%)	HCC	16 (10.5)
	ESC	13 (8.6)
	GC	39 (25.7)
	CRC	6 (3.9)
	LC	78 (51.3)
Stage (%)^a^	1	2 (1.3)
	2	7 (4.6)
	3	30 (19.7)
	4	113 (74.3)
Number of metastases (%)	0	49 (32.2)
	1	56 (36.8)
	2	26 (17.1)
	3	11 (7.2)
	4	9 (5.9)
	5	1 (0.7)
ICI type (%)	Anti-PD1	145 (95.4)
	Anti-PDL1	7 (4.6)
Combination therapy (%)	No	14 (9.2)
	Yes	138 (90.8)
Line of therapy (%)	1	54 (35.5)
	2	46 (30.3)
	3	22 (14.5)
	4	30 (19.7)
Location of target lesions (%)	esophagus	12 (7.9)
	liver	31 (20.4)
	lung	71 (46.7)
	stomach	18 (11.8)
	node	13 (8.6)
	others	7 (4.6)

Data reflect numbers of patients with corresponding percentages in parentheses unless otherwise indicated

*SD* standard deviation, *BMI* body mass index, *HCC* hepatocellular carcinoma, *ESC* esophageal cancer, *GC* gastric cancer, *CRC* colorectal cancer, *LC* lung cancer, *ICI* immune checkpoint inhibitor, *PD1* programmed cell death protein-1, *PDL1* programmed cell death protein ligand-1

^a^defined in accordance with the American Joint Committee on Cancer TNM staging system

### Feature selection and construction of the radiomics signature

**Fig. 2:**
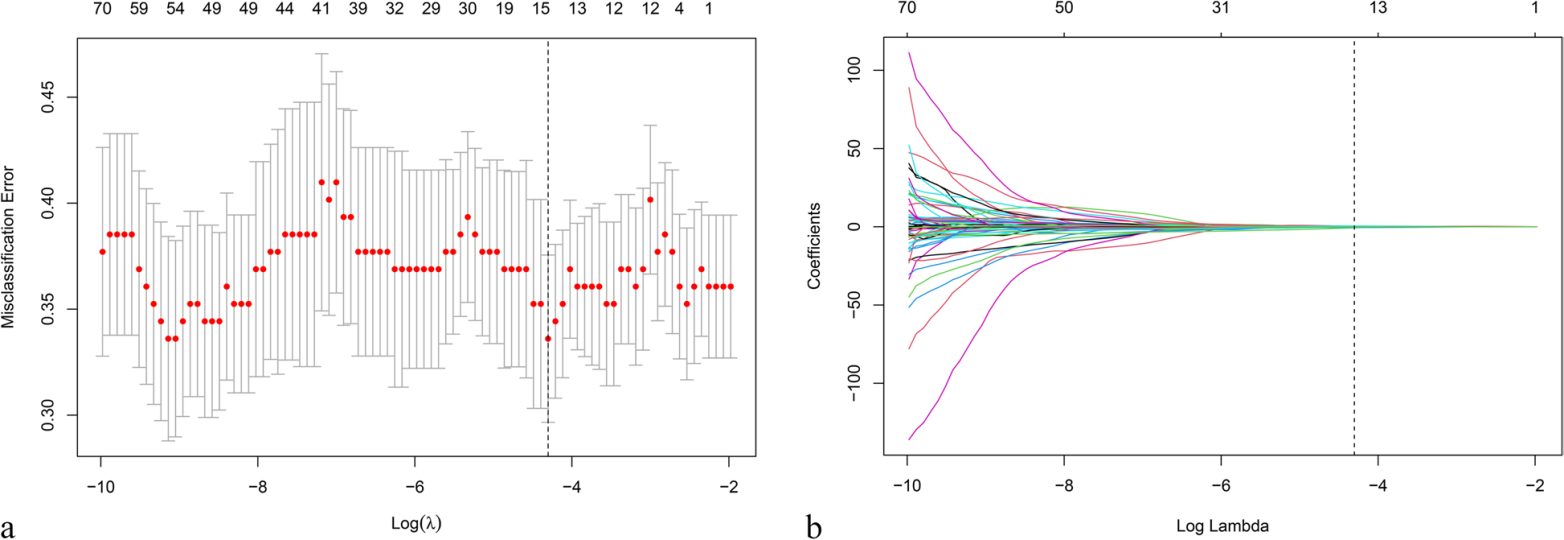
Selection of radiomic features using the least absolute shrinkage and selection operator (LASSO) model**. a** Tuning parameter (λ) selection in the LASSO model using 10-fold cross-validation via minimum criteria. The misclassification errors were plotted against log (λ). The y-axis indicates misclassification errors, the lower x-axis indicates log(λ), and the upper x-axis indicates the degree of freedom (i.e., the number of features surviving at a specific λ). The vertical dotted line shows the optimal values of the tuning parameter (λ) based on the minimum criteria, where the model has the best fit. An λ of 0.01352951 was finally selected. **b** LASSO coefficient profiles of 89 significant features. Coefficient profiles were plotted against the log(λ). The vertical dotted line was drawn at the best λ value selected from the 10-fold cross-validation, and fourteen features with nonzero coefficients were identified

A total of 851 radiomic features were originally extracted from the pretreatment CT scans. During the intra- and inter-observer reliability analyses, 157 features were excluded due to low agreement. Thus, 694 radiomic features remained for selection. After the difference test, 89 features (including target lesion location [liver]) that differed significantly between responders and non-responders in the overall population were retained. Using the training set, the LASSO regression model identified 14 features with nonzero coefficients using an optimal regulation weight (λ) of 0.01352951 based on the minimum criterion (Fig. 2). These 14 features were finally used to construct the radiomics signature, and the radscore was calculated according to the following formula (Additional file Table S2), in which the 14 features were replaced by letters *a* through *n*:

radscore = 0.254 *× a –* 0.027 × *b +* 0.546 × *c* + 0.026 × *d* – 5.469E-11 × *e* – 0.116 × *f* – 0.170×*g +* 0.235 × *h +* 0.318 × *i –* 0.286 × *j –* 0.237 × *k –* 0.664 × *l –* 0.444 × *m –* 0.166 × *n −* 0.706.

Difference analysis indicated that responders had significantly higher radscores than non-responders based on the training and validation sets (Additional file Fig. S1).

### Development of the radiomics nomogram

**Fig. 3:**
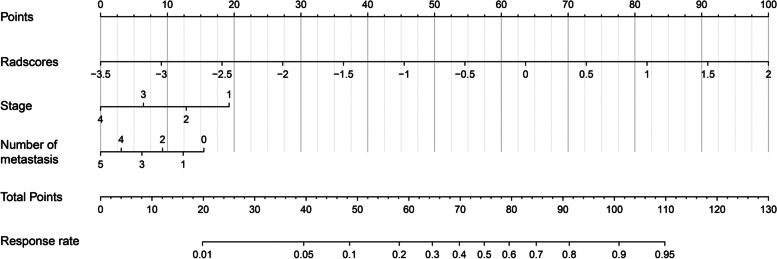
The constructed radiomics nomogram for predicting response to immunotherapy. We developed a radiomics-based nomogram incorporating the radscore, stage, and number of metastases. Radscore: radiomics score

In the overall population, univariate logistic regression indicated that radscore, stage, number of metastases, tumor type (gastric cancer vs. hepatocellular carcinoma), tumor type (lung cancer vs. hepatocellular carcinoma), and line of therapy (> 3 vs. 1) were significantly associated with treatment response (Additional file Table S3). Tumor type and line of therapy were excluded from the multivariate analysis, as not all comparisons of the groups yielded significant results. Using the training set, a radiomics nomogram incorporating the radscore, stage, and number of metastases was developed using multivariate logistic regression (Fig. 3). The following formula was used to calculate each patient’s nomoscore (Additional file Table S4):

nomoscore = 1.523 × radscore – 0.538 × stage – 0.259 × number of metastases + 2.520.

Difference analysis indicated that responders had significantly higher nomoscores than non-responders based on the training and validation sets (Additional file Fig. S2).

### Model performance and validation

**Fig. 4:**
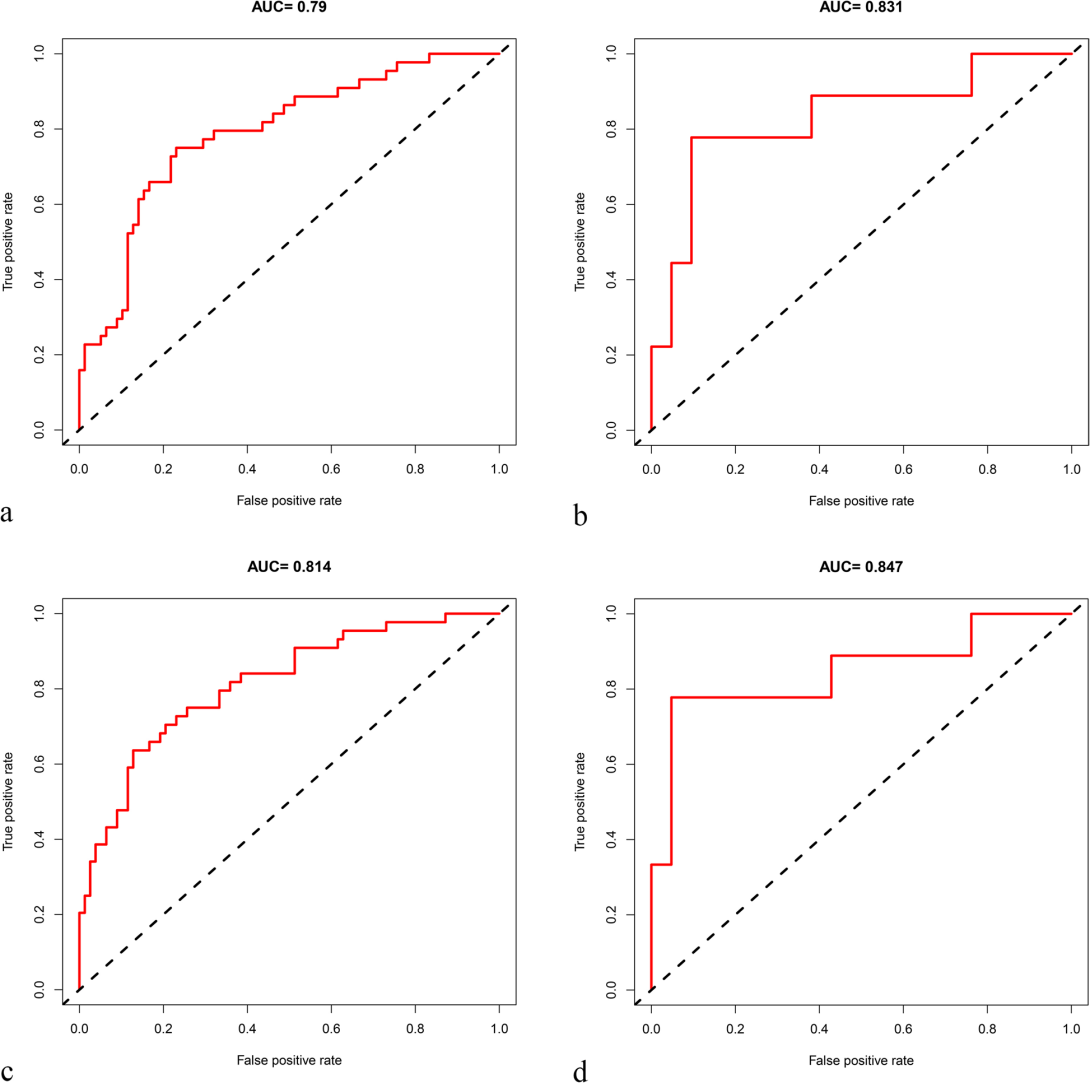
Receiver operating characteristic (ROC) curves for the radiomics score (radscore) and nomogram. Panels **a** and **b** represent the ROC curves for the radscore for the training and validation sets, while panels **c** and **d** represent those for the nomogram for the training and validation sets, respectively. AUC: area under the ROC curve

Calibration evaluation by the Homer–Lemeshow test indicated that there were no evidence of poor fit for two models and both the radscore and nomogram exhibited good accuracy in predicting the treatment response in both the training (radscore, *p* = 0.1532; nomogram, *p* = 0.9576) and validation sets (radscore, *p* = 0.3063; nomogram, *p* = 0.5241). Figure 4 shows the ROC curves for both models based on the training and validation sets. The radscore had AUC values of 0.790 (95% CI [0.705, 0.874]) for the training set and 0.831 (95% CI [0.649, 1]) for the validation set. For the radiomics signature, we also assessed the predictive performance for each included feature individually; the AUC value for a single feature was not favorable (Additional file Table S5). The nomogram had AUC values of 0.814 (95% CI [0.734, 0.893]) for the training set and 0.847 (95% CI [0.662, 1]) for the validation set. The nomoscore exhibited slightly better performance in predicting treatment response than the radscore although the differences were not significant (DeLong test, *p* = 0.1945 in the training set, and *p* = 0.5002 in the validation set). The sensitivities, specificities, and accuracies of the two models, along with their optimal cut-off values, are summarized in Table 2.

**Table 2:** Predictive performance of radiomics scores and nomogram

	Cut-off value^**a**^	AUC [95% CI]	TPR (%)^**b**^	TNR (%)^**b**^	ACC (%)^**b**^
**Radiomics scores**					
Training	−0.478	0.790 [0.705, 0.874]	0.750 (33/44)	0.769 (60/78)	0.762 (93/122)
Validation	−0.478	0.831 [0.649, 1]	0.778 (7/9)	0.810 (17/21)	0.800 (24/30)
**Radiomics nomogram**					
Training	−0.132	0.814 [0.734, 0.893]	0.636 (28/44)	0.872 (68/78)	0.787 (96/122)
Validation	−0.132	0.847 [0.662, 1]	0.778 (7/9)	0.905 (19/21)	0.867 (26/30)

*AUC* area under the receiver operating characteristic curve, *CI* confidence interval, *TPR* true positive rate (i.e., sensitivity), *TNR* true negative rate (i.e., specificity), *ACC* accuracy

^a^using the best threshold determined by the Youden index

^b^calculated according to the cut-off value

### Clinical application

**Fig. 5:**
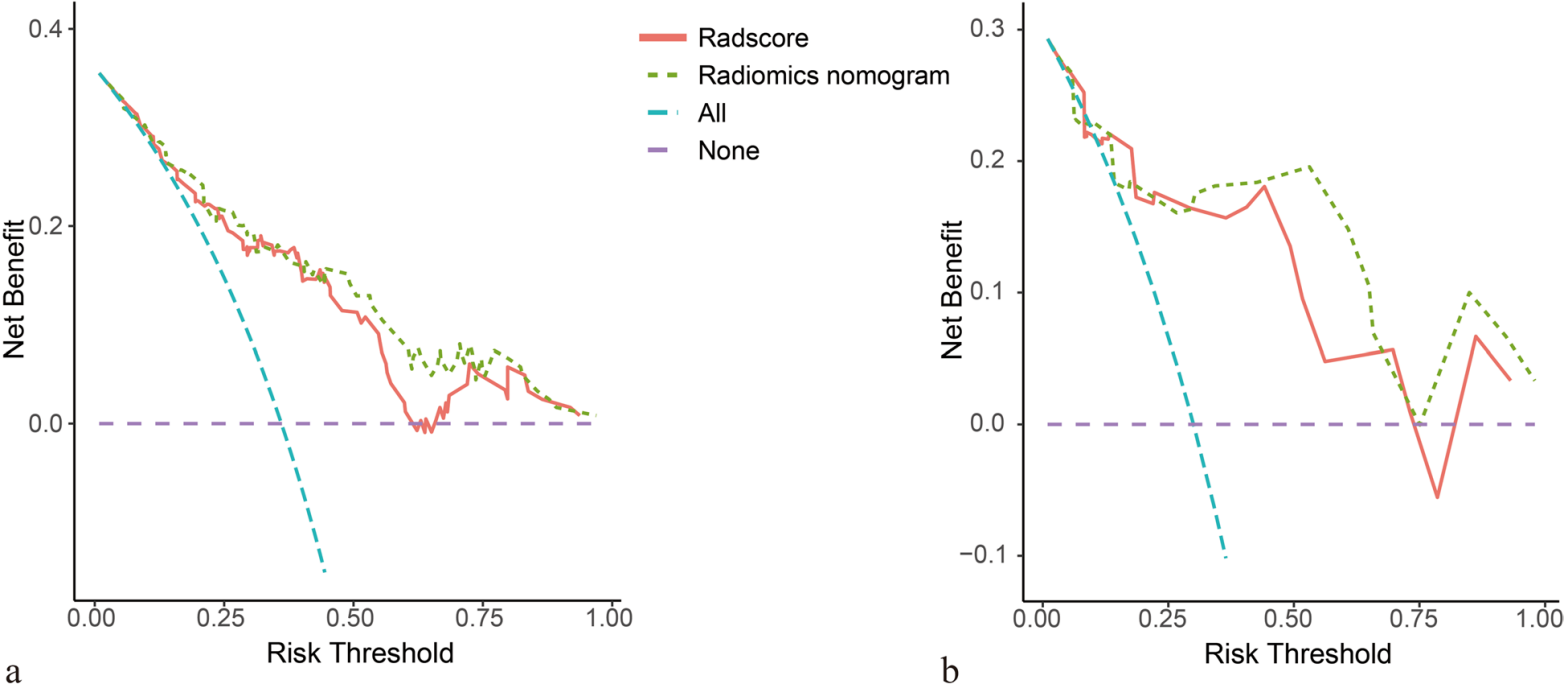
Decision curve analysis (DCA) of the radiomics score (radscore) and nomogram. The x-axis represents the threshold of risk probability, and the y-axis represents the net benefit of the models. The green and purple lines represent the conditions that simply classify all patients as responders or non-responders. DCA revealed a good overall net benefit of the radscore and nomogram for both the training (**a**) and validation sets (**b**), although the net benefit of the nomogram was higher than that of the radscore across the range of reasonable risk thresholds

Decision curve analysis demonstrated a good overall net benefit for the radscore and nomogram for distinguishing responders from non-responders over a wide range of threshold probabilities (Fig. 5). Within a range of reasonable risk thresholds for both the training and validation sets, the nomogram may provide a greater net benefit than the radscore when attempting to predict the response to immunotherapy.

### Association with survival

**Fig. 6:**
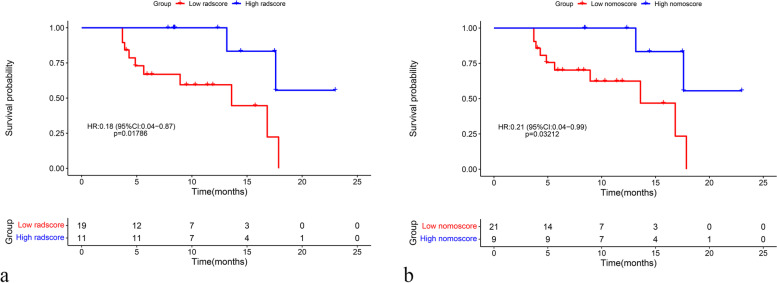
Kaplan–Meier curves for overall survival in the validation set. Significant differences in survival were observed between the low (< cut-off value) and high groups (> cut-off value) in both the radscore and nomoscore analyses. HRs were analyzed when the low group was designated as the control (i.e., high group vs. low group). The corresponding cut-off values were determined by maximizing the Youden index for predicting response. Radscore: radiomics score; Nomoscore: nomogram score; HR: hazard ratio; CI: confidence interval

Survival analysis demonstrated that both the radscore and nomogram were significantly associated with OS (Fig. 6). In the validation set, patients in the high radscore (> − 0.478) group exhibited improved OS when compared with those in the low radscore (≤ − 0.478) group (hazard ratio: 0.18; 95% CI [0.04, 0.87], *p* = 0.01786). Similarly, patients in the high nomoscore (> − 0.132) group also exhibited improved OS when compared with those in the low nomoscore (≤ − 0.132) group (hazard ratio: 0.21; 95% CI [0.04, 0.99], *p* = 0.03212).

## Discussion

As a high proportion of patients exhibit a poor response to immunotherapy, one of the challenges in cancer immunotherapy is identifying the population most likely to benefit from such treatment. Despite substantial effort to develop markers for predicting ICI response, few attempts have been successful. While several studies have reported satisfactory results using clinical factors to predict ICI response [15–18], the robustness of these predictors has been questioned given the high intra- and inter-tumoral heterogeneity. No clinical predictor can fully represent the characteristics of the entire tumor microenvironment, limiting their use in clinical practice. Genetic and protein-based markers are characterized by spatial limitations related to sample collection and cannot fully represent the tumor microenvironment. In the current study, we extracted the radiomic features of the entire tumor from CT images obtained prior to ICI treatment for various types of solid cancer. Although a single feature is not powerful enough to predict the response to ICIs, we successfully developed a radiomics signature for identification of responder by combining fourteen radiomics features, which had an AUC of 0.790 (95% CI [0.705, 0.874]). In addition, we developed a robust radiomics nomogram by combining the radscore with significant clinical variables. According to our analysis, the radiomics nomogram increased performance in discriminating between responders and non-responders, with an AUC of 0.814 (95% CI [0.734, 0.893]).

Both the radscore and nomogram were validated for their performance using the validation set. The results exhibited robust predictive performance, with high AUCs for both models during validation. For a specific evaluation of discriminative performance, both models exhibited satisfactory sensitivity, specificity, and accuracy in the training and validation sets. However, the performance of the radiomics nomogram was superior to that of the radscore, especially for specificity. High specificity is crucial for selecting potential responders to immunotherapy, as misjudgment of responders will increase the financial burden and risk of adverse events among patients. Our nomogram had high specificities of 0.872 and 0.905 for the training and validation sets, respectively, demonstrating the potential clinical usefulness of our models. In addition, both the radscore and nomogram were confirmed an association with OS in the validation set, which further highlights their clinical significance for personalized immunotherapy. In the nomogram model, the variables of stage and metastases number were incorporated with negative coefficients, which indicated both variables are risk factors of poor response. This result is consistent with our conventional perspectives in clinical practice. The stage and number of metastases can reflect the degree of extension of the tumor. With the increase of stage and number of metastases, tumor shows a more aggressive phenotype with higher tumor burden and wider spreading. Thus, it is not hard to explain the finding that both the stage and number of metastases are negatively associated with the response of immunotherapy in cancer patients.

While the ability of the radiomics method to successfully predict the response to ICI treatment remains difficult to explain from a mechanistic perspective, we believe that its predictive ability involves a correlation between radiomic features and the tumor microenvironment. The higher-dimensional data obtained via radiomics may reflect tumor heterogeneity at the cellular level [19, 20]. The effectiveness of immunotherapy is mainly influenced by the status of tumor–immune interactions, and radiomics may represent this characteristic of the tumor microenvironment. Sun et al. identified an association between a radiomics-based signature and immune infiltration in solid tumors [21]. In addition, previous studies have successfully established predictive models for the analysis of tumor PDL-1 expression in various cancers, including non-small cell lung cancer, esophageal squamous cell carcinoma, and head and neck squamous cell carcinoma [22–24]. Both immune infiltration and PDL-1 are key factors involved in cancer immunotherapy; thus, the successful detection of these features on images represents a reasonable means by which radiomics can predict ICI response based on CT images.

Some recent studies have also attempted to predict the response to immunotherapy using radiomics. However, our study has several strengths when compared with these studies. While some radiomics-based models have demonstrated good performance in predicting ICI response, all were developed for lung cancer [25–27], making it difficult to determine whether these models are applicable to other types of cancer. ICI immunotherapy is an intervention with a wide range of applications, and responses are not limited to specific cancers. Thus, a pan-cancer predictor with robust performance across different cancers is required. In our study, we included patients with five different cancer types. Importantly, no cancer-specified variables were included in the final formula, indicating that the constructed models can be used for several cancer types even when the specific type of cancer remains unknown. This is especially appropriate for patients with advanced disease involving metastasis of an unconfirmed pathological type. Ligero et al. also developed models using data from patients with different cancer types [28]. Although their models demonstrated acceptable predictive ability for both the training and validation sets, they were developed using clinical trial data, making them difficult to adopt in clinical practice due to the various confounding factors associated with real-world settings. In addition, they defined response to treatment as complete/partial response or stable disease, which may not be common in crucial evaluations. The features included in our radiomics model differed from those used by Ligero et al. [28], which may be related to differences in the types of images used. In addition to the original images, we also used wavelet-transformed images for feature extraction in our study, as these can provide more information concerning intra-tumor heterogeneity [29].

Our study also had some limitations. First, the sample was relatively small, especially for the validation set. Second, selection bias was inevitable in this study due to its retrospective nature, which might have influenced the results. Third, no external validation was performed owing to the limited number of patients. In addition, radiomics models have not been validated separately for different cancer types. However, we plan to conduct a prospective study to explore the performance and robustness of radiomics models in real-world practice.

## Conclusions

In conclusion, we developed a non-invasive radiomics-based model for early identification of response potential in ICI-treated patients with solid cancers. Radiomics features (with or without clinical factors) were associated with patient response and survival after immunotherapy, thus highlighting the potential of our models to improve clinical decision-making regarding personalized immunotherapy. The results should be adapted into clinical practice with cautions before the further validation from prospective large sample studies.

## Supplementary Information

**Additional file 1: Table S1.** image parameters of CT images in each cohort; **Table S2.** features with their coefficients in the radiomic signature; **Table S3.** univariate logistic regression analysis for association between related variables and immunotherapy response; **Table S4.** variables with their coefficients in the radiomics nomogram; **Table S5.** AUC values for each radiomics feature included in the radiomics signature; **Fig. S1.** Differences in radiomics scores between responders and non-responders; **Fig. S2.** Differences in nomogram scores between responders and non-responders.

## Data Availability

The datasets used and/or analysed during the current study are available from the corresponding author on reasonable request.

## Abbreviations

ICCs: Intra-class correlation coefficients
AUC: Area under the curve
BMI: Body mass index
CI: Confidence interval
ICI: Immune checkpoint inhibitor
LASSO: Least absolute shrinkage and selection operator
OS: Overall survival
PD1: Programmed cell death protein 1
PDL1: Programmed cell death protein ligand 1
RECIST: Response Evaluation Criteria in Solid Tumors
ROC: Receiver operating characteristic
