# Supplementary material for: Radiomics-based prediction of response to immune checkpoint inhibitor treatment for solid cancers using computed tomography: a real-world study of two centers
Source: BMC Cancer. 2022 Nov 30;22:1241. doi: 10.1186/s12885-022-10344-6 (PMC9710011; doi:10.1186/s12885-022-10344-6)
Supplement: Supplementary file 1 — Additional file 1: Table S1. image parameters of CT images in each cohort; Table S2. features with their coefficients in the radiomic signature; Table S3. univariate logistic regression analysis for association between related variables and immunotherapy response; Table S4. variables with their coefficients in the radiomics nomogram; Table S5. AUC values for each radiomics feature included in the radiomics signature; Fig. S1. Differences in radiomics scores between responders and non-responders; Fig. S2. Differences in nomogram scores between responders and non-responders. [file 12885_2022_10344_MOESM1_ESM.docx]

**Radiomics-based prediction of response to immune checkpoint inhibitor treatment for solid cancers using computed tomography: a real-world study of two centers**

**Additional files**

1. **Supplemental tables**

**Table S1 image parameters of CT images in each cohort**

|  | **Level** | | **Institution I (n=128)** | **Institution II (n=24)** |
| --- | --- | --- | --- | --- |
| **Manufacturer (%)** | GE MEDICAL SYSTEMS | | 0 (0.0) | 18 (75.0) |
|  | Philips | | 128 (100.0) | 3 (12.5) |
|  | SIEMENS | | 0 (0.0) | 3 (12.5) |
| **Slice Thickness (mm)** | | (median [IQR])  range | 5.00 [5.00, 5.00]  1.00-5.00 | 5.00 [5.00, 5.00]  1.25-8 |
| **kVp (%)** | 100 | | 0 (0.0) | 2 (8.3) |
|  | 120 | | 128 (100.0) | 22 (91.7) |
| **Convolution Kernel (%)** | B | | 113 (88.3) | 1 (4.2) |
|  | B30f | | 0 (0.0) | 1 (4.2) |
|  | Br40 | | 0 (0.0) | 2 (8.3) |
|  | LUNG | | 0 (0.0) | 1 (4.2) |
|  | STANDARD | | 0 (0.0) | 17 (70.8) |
|  | YB | | 15 (11.7) | 2 (8.3) |
| **Pixel Spacing (mm)** | | (median [IQR])  range | 0.79 [0.75, 0.84]  0.62-0.98 | 0.95 [0.86, 0.98]  0.70-0.98 |

Note: data are numbers of patients with corresponding percentages in parentheses unless otherwise indicated. kVp: kilovoltage peak; IQR, Interquartile range

**Table S2 features with their coefficients in the radiomic signature**

| **Coefficients** | **Features** |
| --- | --- |
| -0.706265717 | (Intercept) |
| 0.253999904 | original_glrlm_ShortRunLowGrayLevelEmphasis |
| -0.027383012 | wavelet.LLH_glcm_Autocorrelation |
| 0.545589797 | wavelet.LLH_glcm_ClusterShade |
| 0.025725972 | wavelet.LLH_glszm_HighGrayLevelZoneEmphasis |
| -5.47E-11 | wavelet.LLH_glszm_LowGrayLevelZoneEmphasis |
| -0.115949453 | wavelet.LHH_glcm_MaximumProbability |
| -0.169546767 | wavelet.LHH_gldm_LargeDependenceLowGrayLevelEmphasis |
| 0.23535349 | wavelet.LHH_glrlm_RunPercentage |
| 0.317813169 | wavelet.LHH_glrlm_ShortRunLowGrayLevelEmphasis |
| -0.286245956 | wavelet.HHH_firstorder_Minimum |
| -0.237279345 | wavelet.HHH_glcm_Imc2 |
| -0.663531527 | wavelet.HHH_glrlm_ShortRunLowGrayLevelEmphasis |
| -0.444159886 | wavelet.LLL_glrlm_HighGrayLevelRunEmphasis |
| -0.165924293 | wavelet.LLL_glrlm_LongRunHighGrayLevelEmphasis |

Note: By using the wavelet transform, original images were decomposed into eight images, which were labeled as LLL, LLH, LHL, LHH, HLL, HLH, HHL and HHH (three letters represent x, y and z axis, respectively; L: low-frequency signals; H: high--frequency signals).

GLRLM: Gray-Level Run Length Matrix; GLCM: Gray Level Co-occurrence Matrix; GLSZM: Gray Level Size Zone Matrix; GLDM: Gray Level Dependence Matrix; lmc2: Informational Measure of Correlation 2

**Table S3 univariate logistic regression analysis for association between related variables and immunotherapy response**

| **Variables** | **OR value** | **95% CI** | **P value** |
| --- | --- | --- | --- |
| **Radscores** | 4.65 | [2.65, 8.17] | 8.85E-08 |
| **Sex (female vs male)** | 0.43 | [0.17, 1.06] | 0.067774 |
| **Age** | 1 | [0.97, 1.03] | 0.998309 |
| **BMI** | 1.02 | [0.93, 1.12] | 0.702221 |
| **Tumor type (ESC vs HCC)** | 9.37 | [0.93, 94.65] | 0.057807 |
| **Tumor type (GC vs HCC)** | 9.37 | [1.12, 78.46] | 0.038953 |
| **Tumor type (CRC vs HCC)** | 3 | [0.16, 57.37] | 0.465566 |
| **Tumor type (LC vs HCC)** | 9.89 | [1.24, 78.75] | 0.030353 |
| **Stage** | 0.47 | [0.27, 0.81] | 0.00671 |
| **Number of metastasis** | 0.7 | [0.51, 0.96] | 0.028328 |
| **ICI type (anti-PDL1 vs anti-PD1)** | 2.61 | [0.56, 12.14] | 0.22048 |
| **Combination therapy (yes vs no)** | 0.96 | [0.3, 3.03] | 0.944435 |
| **Line of therapy (2 vs 1)** | 0.95 | [0.43, 2.1] | 0.8965 |
| **Line of therapy (3 vs 1)** | 0.4 | [0.13, 1.23] | 0.109666 |
| **Line of therapy (>3 vs 1)** | 0.34 | [0.12, 0.96] | 0.041254 |

Note: OR: odds ratio; CI: confidence interval; radscores: Radiomics scores; BMI: : body mass index; HCC: hepatocellular carcinoma; ESC: esophageal cancer; GC: gastric cancer; CRC: colorectal cancer; LC: lung cancer; ICI: immune checkpoint inhibitor; PD1: programmed cell death protein–1; PDL1: programmed cell death protein ligand–1. P<0.05 was considered statistically significant.

**Table S4 variables with their coefficients in the radiomics nomogram**

| **Coefficients** | **Variables** |
| --- | --- |
| 2.5198 | Intercept |
| 1.5234 | Radscores |
| -0.5375 | Stage |
| -0.2591 | Number of metastasis |

Note: Radscores: radiomics scores

**Table S5 AUC values for each radiomics feature included in the radiomics signature**

| **Features** | **AUC [95% CI]** | |
| --- | --- | --- |
|  | **Train set** | **Validation set** |
| original_glrlm_ShortRunLowGrayLevelEmphasis | 0.671 [0.572, 0.77] | 0.529 [0.292, 0.766] |
| wavelet.LLH_glcm_Autocorrelation | 0.355 [0.256, 0.455] | 0.302 [0.085, 0.518] |
| wavelet.LLH_glcm_ClusterShade | 0.615 [0.513, 0.717] | 0.698 [0.477, 0.919] |
| wavelet.LLH_glszm_HighGrayLevelZoneEmphasis | 0.616 [0.513, 0.718] | 0.667 [0.456, 0.877] |
| wavelet.LLH_glszm_LowGrayLevelZoneEmphasis | 0.384 [0.282, 0.487] | 0.333 [0.123, 0.544] |
| wavelet.LHH_glcm_MaximumProbability | 0.418 [0.313, 0.523] | 0.376 [0.15, 0.602] |
| wavelet.LHH_gldm_LargeDependenceLowGrayLevelEmphasis | 0.392 [0.29, 0.495] | 0.392 [0.175, 0.608] |
| wavelet.LHH_glrlm_RunPercentage | 0.622 [0.521, 0.723] | 0.529 [0.294, 0.765] |
| wavelet.LHH_glrlm_ShortRunLowGrayLevelEmphasis | 0.632 [0.532, 0.732] | 0.593 [0.37, 0.815] |
| wavelet.HHH_firstorder_Minimum | 0.437 [0.325, 0.55] | 0.455 [0.135, 0.775] |
| wavelet.HHH_glcm_Imc2 | 0.391 [0.286, 0.496] | 0.354 [0.135, 0.574] |
| wavelet.HHH_glrlm_ShortRunLowGrayLevelEmphasis | 0.611 [0.509, 0.714] | 0.503 [0.26, 0.745] |
| wavelet.LLL_glrlm_HighGrayLevelRunEmphasis | 0.453 [0.411, 0.494] | 0.429 [0.352, 0.505] |
| wavelet.LLL_glrlm_LongRunHighGrayLevelEmphasis | 0.402 [0.3, 0.504] | 0.45 [0.236, 0.663] |

Note: AUC: area under the ROC curve; CI: confidence interval

1. **Supplemental figures**

**
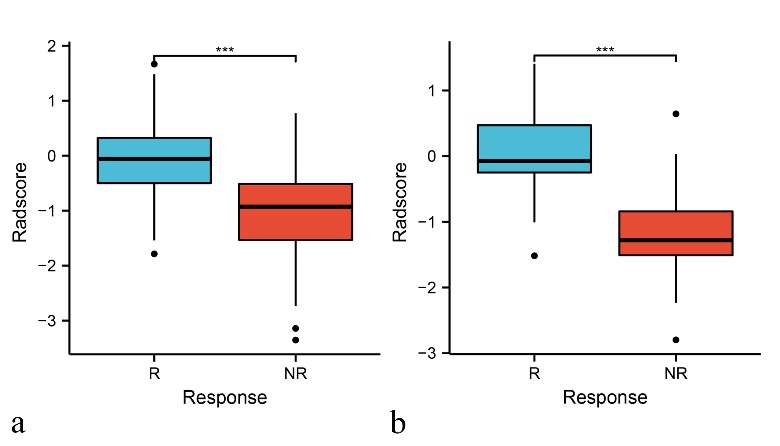
**

**Figure S1 Differences in radiomics scores between responders and non-responders.** Boxplots show that radscores were significantly higher for responders than for non-responders in the training (a) and validation (b) sets. Radscore: radiomics score; R: responders; NR: non-responders. *** indicates p<0.001.


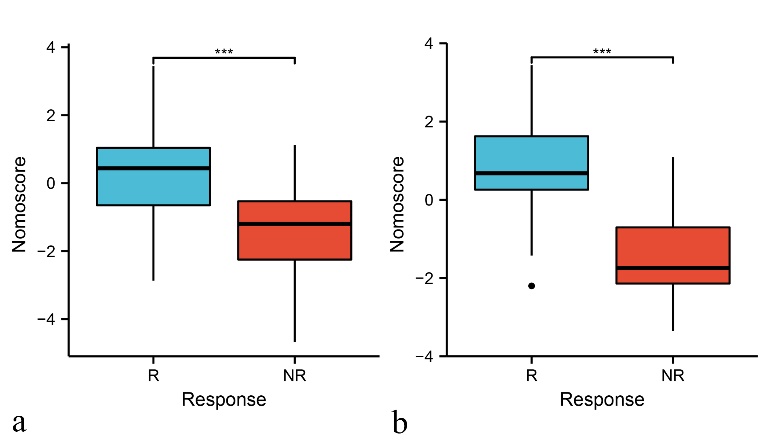


**Figure S2 Differences in nomogram scores between responders and non-responders.** Boxplots show that nomoscores were significantly higher for responders than for non-responders in the training (a) and validation (b) sets. Nomoscore: nomogram score; R: responders; NR: non-responders. *** indicates p<0.001.
